# A Rare Presentation in a Common Renal Anomaly: Metastatic Adenocarcinoma in a Horseshoe Kidney

**DOI:** 10.7759/cureus.81936

**Published:** 2025-04-09

**Authors:** Mariam Malik, Rana Bilal Idrees, Maryam Ikram, Maham Khalid, Muhammad Hamid Chaudhary

**Affiliations:** 1 Radiology, Atomic Energy Cancer Hospital, Nuclear Medicine, Oncology and Radiotherapy Institute (NORI), Islamabad, PAK; 2 Radiology, Institute of Nuclear Medicine and Oncology Lahore (INMOL) Cancer Hospital, Lahore, PAK; 3 Diagnostic Radiology Department, Institute of Nuclear Medicine and Oncology Lahore (INMOL) Cancer Hospital, Lahore, PAK; 4 Cardiac Surgery, Chaudhry Pervaiz Elahi Institute of Cardiology, Multan, PAK

**Keywords:** dorsal vertebrae, horseshoe kidney, metastasis, renal fusion anomalies, renal pelvis adenocarcinoma

## Abstract

Horseshoe kidney is the most common renal fusion anomaly, occurring in approximately 1 in 500 individuals. While renal cell carcinoma (RCC) is the most frequently reported malignancy in horseshoe kidneys, primary adenocarcinoma of the renal pelvis is exceedingly rare, accounting for less than 1% of all renal malignancies. Due to its rarity and nonspecific presentation, diagnosis is often delayed, leading to poor prognosis.

We report the case of a 35-year-old male who presented with progressive abdominal pain and backache for three months. Initial imaging, including radiography, ultrasound, and contrast-enhanced computed tomography (CT), revealed a horseshoe kidney with a heterogeneously enhancing mass arising from the left renal pelvis, containing coarse calcifications. Further evaluation with magnetic resonance imaging (MRI) and fluorodeoxyglucose (FDG) positron emission tomography (PET) demonstrated metastatic involvement of the dorsal vertebrae (D4, D8) and first lumbar vertebra (L1), along with pulmonary metastases. CT-guided biopsy confirmed primary adenocarcinoma of the renal pelvis. Next-generation sequencing (NGS) did not reveal actionable mutations, limiting the role of targeted therapy. The patient was managed with systemic chemotherapy and palliative radiotherapy for bone metastases. This case underscores the importance of multimodal imaging for accurate diagnosis and staging, as well as the role of molecular profiling in treatment planning. Given the poor prognosis associated with metastatic disease, further research into the molecular characteristics and targeted therapies for this rare malignancy is warranted.

## Introduction

Horseshoe kidney is the most common renal fusion anomaly, affecting about 1 in 500 individuals [1]. It results from the abnormal fusion of the lower poles of the kidneys during fetal development, leading to a U-shaped kidney located in the midline, anterior to the great vessels. While the majority of individuals with horseshoe kidneys remain asymptomatic, this anatomical variation predisposes them to a higher risk of urological complications, including urinary tract infections, hydronephrosis, nephrolithiasis, and, more rarely, malignancies.

Malignancies in horseshoe kidneys have rarely been reported of which renal cell carcinoma (RCC) is the most common [2]. Other neoplasms, such as transitional cell carcinoma (TCC) of the renal pelvis, have also been reported, albeit less frequently. Primary adenocarcinoma of the renal pelvis accounts for less than 1% of all renal malignancies [3] and has seldom been reported given the rarity of this condition and its association with poor prognosis. The presence of a horseshoe kidney in this context further adds to its unique occurrence.

In the present case, a young male with a horseshoe kidney presented with back pain and was found to have a left renal pelvic mass with distant vertebral and pulmonary metastases. The diagnosis was confirmed through a CT-guided biopsy of the renal mass, as well as review biopsies from the lung and vertebral lesions, which demonstrated metastatic morphology consistent with the primary tumor. Histopathological examination revealed malignant glandular architecture with complex branching, irregular, and tubular structures. The immunohistochemical (IHC) profile showed positivity for cytokeratin (CK) and carcinoembryonic antigen (CEA), with negativity for markers typically associated with RCC (e.g., PAX8, CD10) and urothelial carcinoma (e.g., GATA3, uroplakin). This supported the diagnosis of primary adenocarcinoma of the renal pelvis. Given the rarity and aggressive nature of this tumor, this report highlights the value of comprehensive imaging, histopathological correlation, and careful differential diagnosis in such atypical presentations.

## Case presentation

A 35-year-old male presented to our clinic with a three-month history of worsening abdominal pain and backache. The pain was insidious in onset, dull in nature, and localized to the left flank, as well as the mid and lower back. It was not associated with fever, weight loss, hematuria, or urinary symptoms. The patient denied any significant past medical history, including urological issues, and there was no known family history of malignancies.

On physical examination, the patient appeared in mild distress due to pain. Abdominal examination revealed tenderness in the left flank with a palpable mass. Neurological examination of the spine was normal, with no signs of motor or sensory deficits.

**Figure 1:**
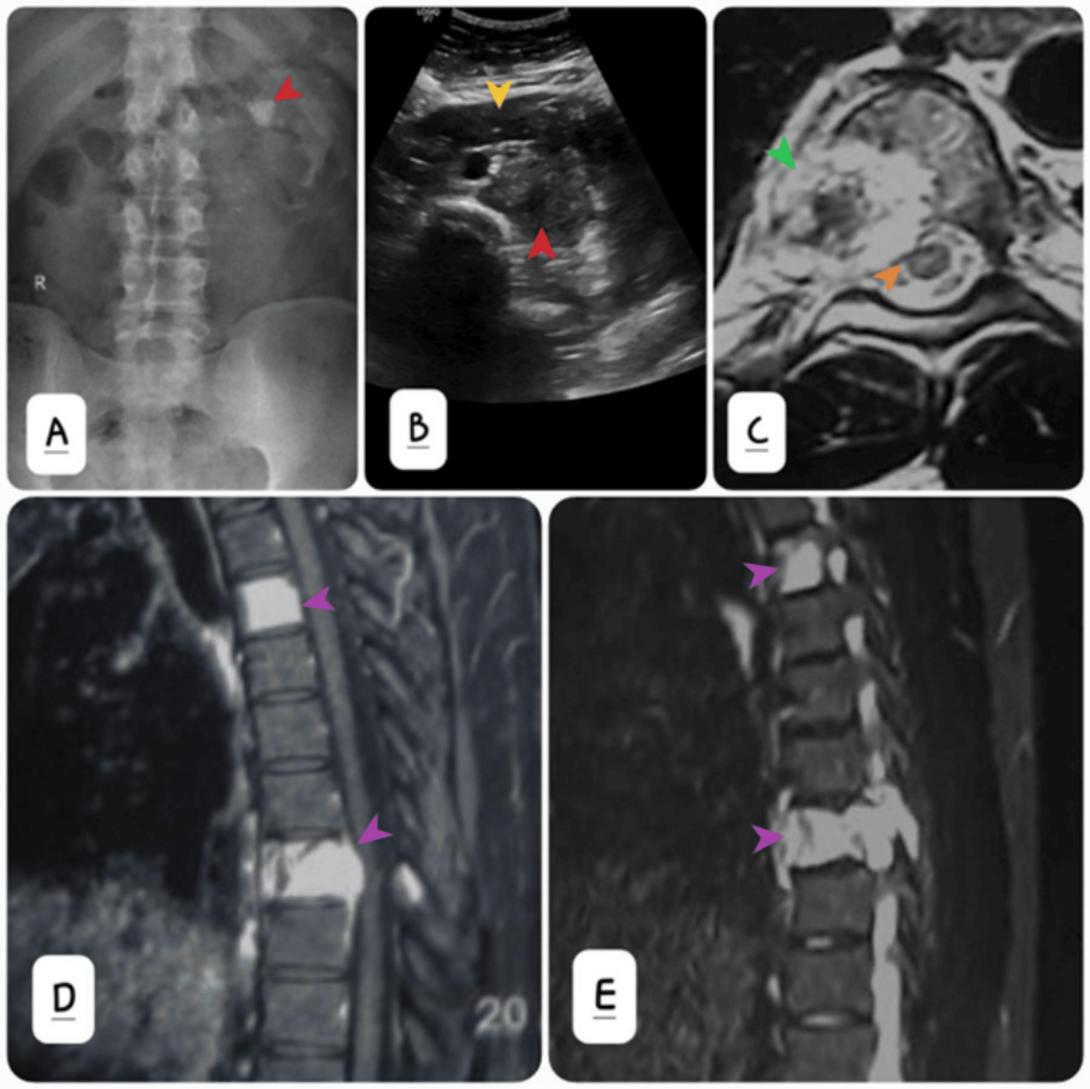
(A) Plain radiograph abdomen showing calcification in the mass at the left renal fossa (red arrow). (B) Transverse grey scale ultrasound showing horseshoe kidneys (yellow arrow) and left renal mass (red arrow). (C) Axial T2W MRI image showing osteolytic lesion (green arrow) in D8 vertebra with intraspinal extension of soft tissue component (orange arrow). (D) Post-contrast sagittal T1W and (E) Sagittal T2W MRI images showing vertebral metastasis (purple arrows).

Routine laboratory investigations were unremarkable, including normal renal function tests and a complete blood count revealing mild normocytic anemia. Urinalysis showed microscopic hematuria but no evidence of infection or pyuria. The patient was advised to have a radiograph of the lumbar spine and an ultrasound abdomen as a part of the initial work-up. The anterior-posterior (AP) view of the radiograph lumbar spine showed low-lying kidneys that appeared horseshoe-shaped with chunky macro calcification in the left lumbar region (Figure 1A). Ultrasound confirmed a horseshoe kidney with a hyperechoic mass arising from the left renal pelvis (Figure 1B). Magnetic resonance imaging (MRI) thoracolumbar spine with contrast was also advised for the work-up of backache (Figures 1C-1E). There were altered signal intensity lesions in the fourth and eighth dorsal vertebrae (D4 and D8) as well as the first lumbar vertebra (L1) with associated soft tissue components.

**Figure 2:**
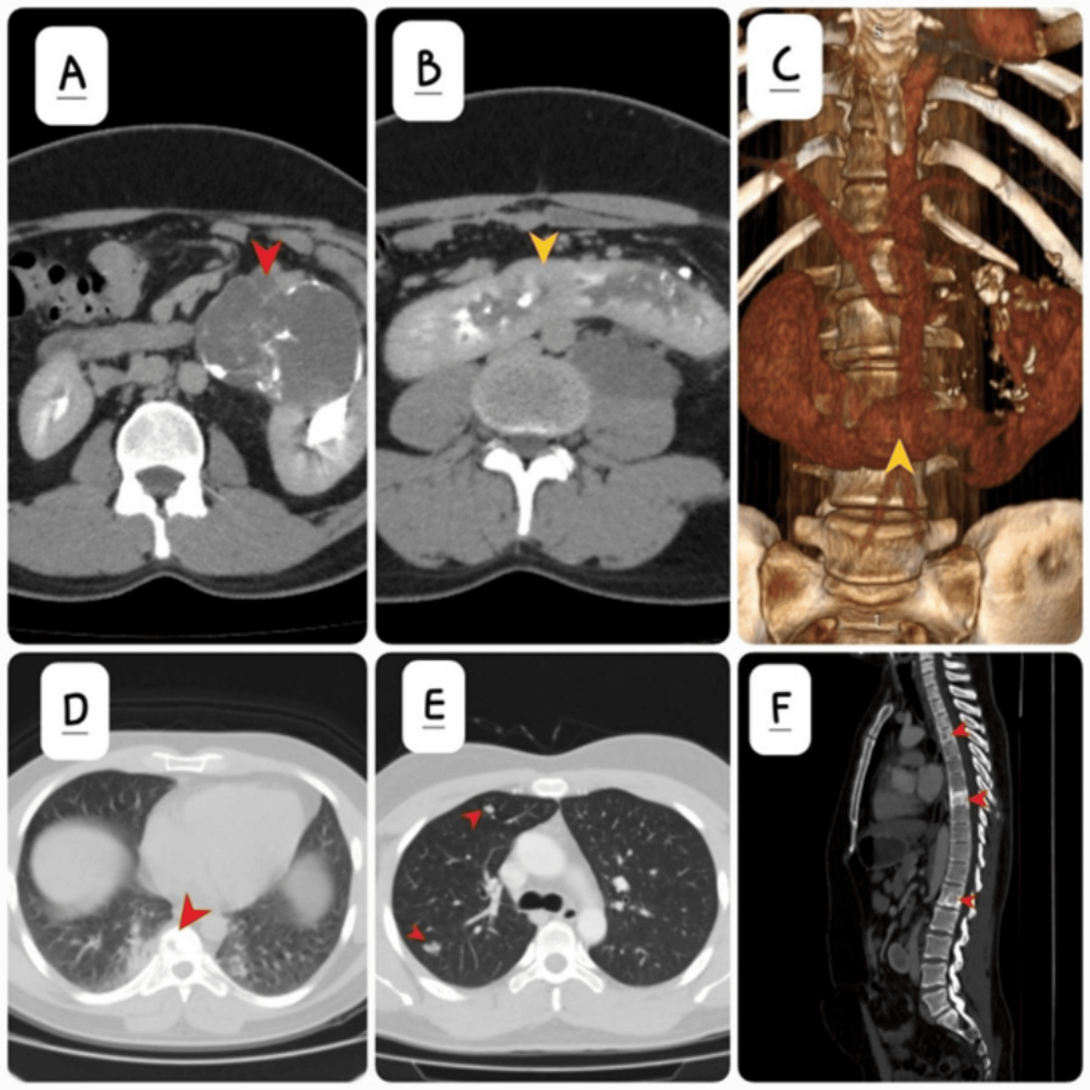
(A) Axial contrast-enhanced CT (CECT) scan showing left renal mass with multifocal coarse calcification (red arrow). (B) Axial CECT demonstrates the horseshoe kidney with yellow arrow annotating the stump. (C) 3D volume rendered coronal image again demonstrates the horseshoe kidney and dense foci of calcification in the left lumbar fossa. (D) Axial CECT image with a lytic lesion in the thoracic vertebra (red arrow). (E) Soft tissue pulmonary nodules suspicious for metastasis (red arrows). (F) Sagittal CT section, a bone window that shows osteoblastic lesions in the fourth and eighth dorsal vertebrae (D4 and D8) as well as the first lumbar vertebra (L1) (red arrows).

A contrast-enhanced computed tomography (CT) scan of the abdomen and pelvis was performed (Figures 2A-2F), which revealed the presence of a horseshoe kidney with fusion of the lower poles across the midline. A heterogeneously enhancing mass, approximately 6 cm in diameter, was identified in the left renal pelvis, with irregular margins and extension into the adjacent renal parenchyma. The mass extended posteriorly into the left para-aortic space. Intralesional foci of calcification were also evident. There was evidence of metastasis to the fourth and eighth dorsal vertebrae (D4 and D8) as well as the first lumbar vertebra (L1) confirmed by multiple osteoblastic lesions in the spine. Soft tissue nodules were seen in bilateral lung parenchyma suspicious for metastasis. No liver or nodal metastases were observed.

**Figure 3:**
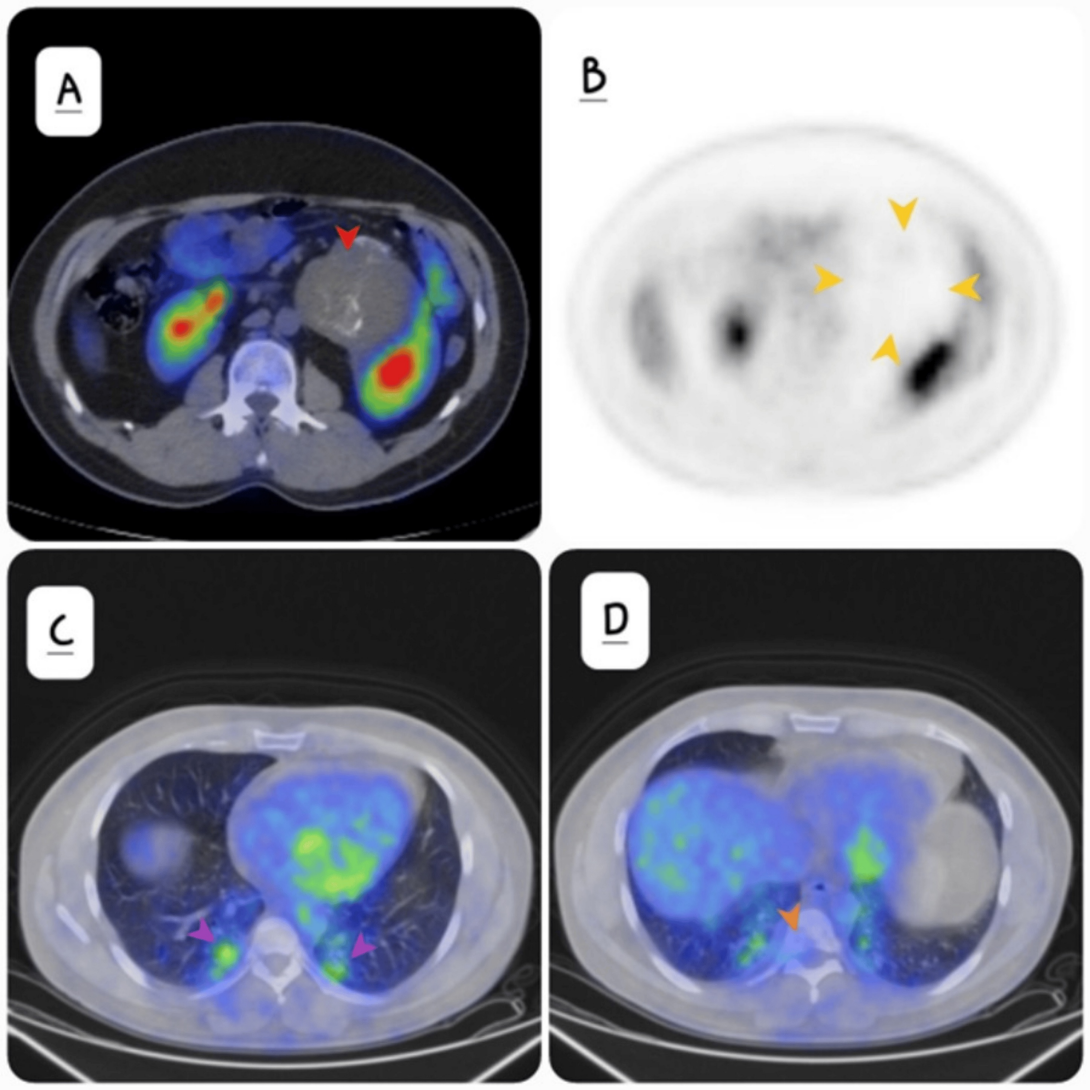
(A) Axial tomographic FDG PET-CT fusion image reveals the mass is hypo metabolic (red arrow). B) PET tomographic axial image confirms the mass is photopenic (yellow arrows). (C) Shows hypermetabolic lung metastasis (purple arrows) and (D) demonstrates hypermetabolic bone metastasis (orange arrow) FDG: Fluorodeoxyglucose

Fluorodeoxyglucose (FDG) positron emission tomography (PET) CT showed the renal mass was hypo metabolic (Figure 3A) and appeared photopenic (Figure 3B). There were hypermetabolic metastatic deposits in the lung parenchyma (maximum SUV value 3.1 with reference to maximum SUV liver of 2.4). There were hypermetabolic lesions in the fourth and eighth dorsal vertebrae (D4 and D8) as well as the first lumbar vertebra (L1) (maximum SUV 3.4 with reference to maximum SUV liver of 2.4) (Figures 3C-3D). 

**Figure 4:**
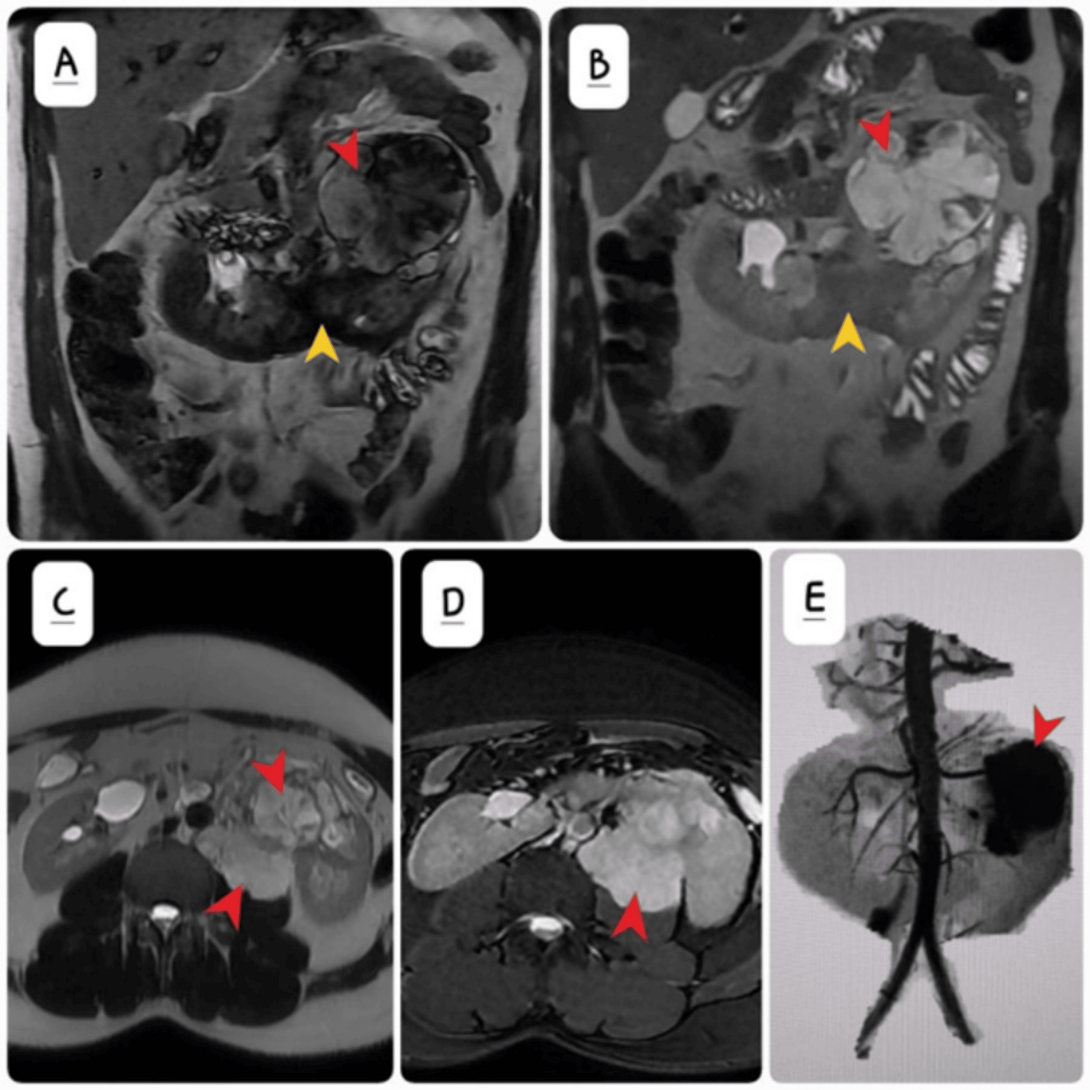
(A) Coronal T1 non-contrast shows the mass returns heterogeneous signals (red arrow). The stump of the horseshoe kidney is indicated by yellow arrow. (B) Coronal T2W and (C) axial T2W shows predominant hyperintense signal mass arising from the left renal pelvis (red arrows). (C) Axial T2 fat-suppressed image demonstrating the mass is hyperintense (red arrow). (E) Time-of-flight magnetic resonance angiography (MRA) shows intense neovascularity arising from the left renal artery. Mass is annotated by red arrow.

MRI abdomen without contrast was also done (Figures 4A-4E) which reiterated the findings of CECT. 

The imaging and metastatic findings have been summarized in a concise table for quick reference (Table 1). 

**Table 1:** Imaging modality and metastatic findings FDG: Fluorodeoxyglucose

Imaging Modality	Findings
Radiography (AP View)	Low-lying kidneys with a horseshoe configuration; coarse calcification in the left lumbar region.
Ultrasound	Horseshoe kidney with a hyperechoic mass originating from the left renal pelvis.
Contrast-enhanced CT	Heterogeneously enhancing 6 cm mass in the left renal pelvis with coarse calcifications; extension into adjacent renal parenchyma and posteriorly into the left para-aortic space; metastases in D4, D8, L1 vertebrae; bilateral pulmonary nodules suggestive of metastases.
MRI (Thoracolumbar spine)	Altered signal intensity lesions in D4, D8, and L1 vertebrae with associated soft tissue components, indicative of metastatic involvement.
FDG PET-CT	Hypometabolic renal mass (photopenic); hypermetabolic metastatic lesions in lung parenchyma (SUV max 3.1) and D4, D8, L1 vertebrae.

**Figure 5:**
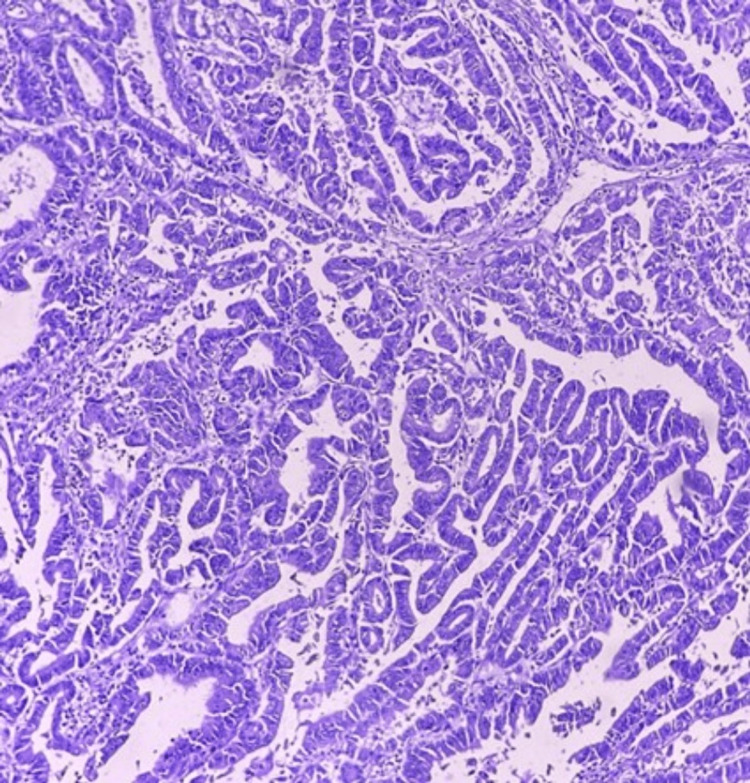
H&E histopathology image demonstrating a glandular pattern with complex, branching, and irregular tubular structures consistent with adenocarcinoma. The glands are lined by atypical epithelial cells showing hyperchromatic nuclei, prominent nucleoli, and nuclear pleomorphism. Mitotic figures are visible, indicating active cell proliferation. The surrounding stroma appears desmoplastic, suggesting an invasive process.

A CT-guided percutaneous biopsy of the renal mass was performed. Histopathological analysis confirmed the diagnosis of adenocarcinoma originating from the renal pelvis (Figure 5). An extended immunohistochemical panel was employed to establish the diagnosis. The tumor cells showed positivity for cytokeratin 7 (CK7) and CEA, indicating glandular differentiation. Notably, markers typically associated with RCC (PAX8, CD10) and urothelial carcinoma (GATA3, uroplakin) were negative, supporting a diagnosis of primary adenocarcinoma. Given the uncommon presentation of primary adenocarcinoma of the renal pelvis with widespread metastases, the possibility of dual primaries was considered. To address this, image-guided biopsies of both pulmonary and vertebral lesions were performed. Histopathological analysis of these metastatic sites revealed features consistent with adenocarcinoma, similar to those observed in the renal mass. Additional markers such as thyroid transcription factor 1 (TTF-1) and napsin A were also negative in lung and bone biopsies, effectively ruling out a lung primary and confirming metastatic spread from the renal lesion. These findings confirmed that the lung and bone lesions represented metastatic spread from the primary renal pelvic adenocarcinoma rather than separate primary malignancies. This comprehensive approach effectively ruled out the presence of dual primaries in this patient. Due to institutional rules and regulations, the IHC images cannot be provided for research purposes and hence the images are not included in the case report. The biopsy results were re-reviewed at an international lab where NGS was also conducted. The results concluded similar findings and in addition, next-generation sequencing (NGS) tested negative. An elaborated IHC table is shown (Table 2) for quick referencing. 

**Table 2:** Immunohistochemical markers for differential diagnosis of renal, urothelial, and metastatic tumors TCC: transitional cell carcinoma; RCC: renal cell carcinoma; TTF-1: thyroid transcription factor 1

Marker	Typical Expression	Utility
CK7	Positive in adenocarcinomas (incl. upper tract)	Supports adenocarcinoma or urothelial origin
CEA	Positive in gland-forming adenocarcinomas	Suggests glandular differentiation
PAX8	Positive in RCC, negative in most urothelial and GI tumors	Helps distinguish RCC from others
GATA3	Positive in urothelial carcinoma	Negative in most adenocarcinomas
CD10	Commonly positive in RCC	Helps support RCC if present
CK20	Variable; may help in GI adenocarcinomas	Adds specificity in context
Villin/MUC markers	May help if considering GI metastasis	Use if clinical suspicion exists
TTF-1/Napsin A	Positive in primary lung adenocarcinoma	Important to rule out lung primary in mets
Uroplakin	Positive in urothelial carcinoma	Helps exclude TCC if negative

The patient was started on six cycles of combination chemotherapy with gemcitabine and cisplatin which was given every 3 weeks. Ten sessions of radiotherapy were also given for vertebral metastasis for consecutive 10 days to alleviate pain. After three cycles of chemotherapy follow-up CT chest abdomen pelvis showed disease regression. The patient is presently post four cycles of chemotherapy and 10 sessions of radiotherapy and is doing better on follow-up.

## Discussion

Adenocarcinoma of the renal pelvis has unique diagnostic complexities, especially in the context of a horseshoe kidney. It often arises from chronic inflammatory conditions, such as recurrent pyelonephritis, renal calculi, or hydronephrosis, which lead to metaplasia of the urothelial cells lining the renal pelvis [4]. The pathogenesis involves prolonged exposure to irritants, resulting in mucinous metaplasia and subsequent malignant transformation into adenocarcinoma [5]. This case is particularly unusual due to the involvement of a horseshoe kidney, a congenital anomaly that is rarely associated with primary adenocarcinoma.

Horseshoe kidney has been linked to a variety of renal malignancies, with RCC being the most commonly reported [6]. However, adenocarcinoma in the context of horseshoe kidney is exceedingly rare. The congenital fusion of the kidneys and the abnormal vascular supply and drainage may predispose individuals to urological complications, including malignancies. Chronic stasis of urine and recurrent infections are thought to play a significant role in the carcinogenesis process within the renal pelvis. Furthermore, the abnormal positioning of the horseshoe kidney can make early detection of tumors more challenging, often resulting in a delayed diagnosis and presentation at an advanced stage, as seen in this case.

Ultrasound is usually the first investigation for evaluation of abdominal symptoms that can demonstrate a well-circumscribed to ill-defined solid-appearing hypoechoic lesion with internal calcification and necrosis. Contrast-enhanced CT scan with renal mass protocol is the modality of choice for the evaluation of renal masses and disease staging [7]. The tumor appears isodense to hypodense on unenhanced study with intralesional coarse calcification and demonstrates post-contrast enhancement. There is almost always necrosis within the lesion and larger masses appear heterogenous. In this case, advanced imaging provided crucial insights: CT revealed a calcified, necrotic mass in the renal pelvis and vertebral metastases, underscoring the tumor’s aggressive nature. For patients eligible for surgical resection, CT angiography is advocated to see for renal vascularity (which can be variable in a horseshoe kidney) [8] and this further aids in pre-operative planning. Alternatively, an MRI abdomen with contrast can also be used for evaluation of tumors arising in a horseshoe kidney in which case mucinous adenocarcinoma would return T1 hypointense, and T2 hyperintense signals with post-contrast enhancement. FDG PET-CT allows for the evaluation of distant metastasis.

The metastatic spread to the dorsal vertebrae in this patient is also of particular interest, as bone metastases are less commonly observed in primary adenocarcinoma of the renal pelvis. Skeletal metastases in renal malignancies are usually associated with RCC, and the presence of vertebral metastases in this case indicates the aggressive nature of the disease. Vertebral involvement is often associated with significant morbidity, including pain, spinal instability, and neurological deficits, though our patient did not exhibit neurological symptoms at presentation. The evaluation of osseous metastasis can be done by a bone scan.

In this case, the patient’s persistent back pain corresponded with imaging findings of metastatic lesions in the dorsal (D4, D8) and lumbar (L1) vertebrae. This correlation underscores the importance of thorough evaluation of back pain in oncology patients, as spinal metastases can manifest primarily through such symptoms. Pain is the most common presenting complaint in patients with spinal metastases, often being the initial symptom that prompts further investigation. Therefore, in patients with known malignancies who present with new or worsening back pain, it is crucial to assess for possible spinal metastatic involvement to guide appropriate management.

The NGS was performed to address the potential actionable mutations. NGS results were negative for identifiable mutations in genes commonly associated with renal malignancies such as Von Hippel-Lindau (VHL), tumor protein p53 (TP53), and mesenchymal-epithelial transition factor (MET). For non-specialist readers, negative NGS findings mean that no common genetic mutations or alterations known to guide targeted therapy were identified in this tumor. In clinical terms, this suggests that currently available molecular-based treatments may not be applicable in this case. This doesn’t mean there are no treatment options. It just means more detailed testing such as comprehensive genomic profiling (CGP) for other genetic changes might be needed in the future to look for less common changes that could help guide care. It is possible that CGP, which evaluates a broader range of genes and molecular alterations, could provide additional insights. Given the rarity and aggressive nature of metastatic adenocarcinoma of the renal pelvis, CGP might uncover uncommon mutations, gene fusions, or alterations in pathways not typically included in standard panels. These findings could have potential diagnostic, prognostic, or therapeutic implications, and may guide future personalized treatment approaches or clinical trial eligibility.

The absence of mutations limited the use of targeted therapies (immunotherapy), such as tyrosine kinase inhibitors or mammalian target of rapamycin (mTOR) inhibitors, which are often used in RCC. While the significance of negative NGS in primary adenocarcinoma of the renal pelvis is not fully understood due to the rarity of this tumor, the findings suggest that systemic chemotherapy remains the most appropriate treatment option in the absence of identifiable genetic targets. This highlights the need for further research into the molecular characteristics of this rare tumor. Moreover, given the rarity of adenocarcinoma of the renal pelvis, there is a lack of literature specifically addressing its mutational profile, underscoring the need for future research to explore its molecular landscape and identify potential therapeutic targets.

Management of adenocarcinoma of the renal pelvis typically involves surgical resection, particularly nephroureterectomy in localized disease [9]. However, in cases with metastatic involvement, as in this patient, systemic chemotherapy, targeted therapy, or immunotherapy are considered the mainstays of treatment. Given the patient’s advanced disease with vertebral metastases, surgery was not considered a viable option, and the patient was started on systemic chemotherapy with palliative intent. Radiation therapy was recommended for the dorsal vertebral metastases to alleviate pain and reduce the risk of spinal cord compression.

The prognosis for adenocarcinoma of the renal pelvis is generally poor [10]. The five-year survival rate for patients with metastatic renal pelvic carcinoma is significantly reduced compared to those with localized disease. In this case, the presence of distant metastasis to the spine further worsened the prognosis. The patient was counseled regarding the nature of his disease, and regular follow-up was planned to monitor his response to chemotherapy and assess for any complications from the vertebral metastases.

## Conclusions

This case underscores the rare occurrence of primary adenocarcinoma of the renal pelvis in a horseshoe kidney, highlighting the complexities in diagnosis and management. The atypical presentation, coupled with the anatomical challenges of a horseshoe kidney, often leads to delayed detection, allowing for advanced disease progression, as seen in this patient with vertebral and pulmonary metastases. Multimodal imaging played a pivotal role in characterizing the tumor and assessing its metastatic spread, while histopathology confirmed the diagnosis. The absence of actionable mutations on NGS limited targeted therapeutic options, reinforcing the need for further molecular research in this rare malignancy. Given the advanced stage, systemic chemotherapy and palliative radiotherapy were pursued, as curative resection was not feasible. The poor prognosis associated with metastatic adenocarcinoma of the renal pelvis emphasizes the critical need for early detection strategies, individualized treatment approaches, and the exploration of novel targeted therapies to improve patient outcomes.
